# Clinical characteristics and outcomes of severe *Legionella pneumophila* pneumonia diagnosed by metagenomic next-generation sequencing in children: a case series of 8 patients

**DOI:** 10.3389/fcimb.2026.1865333

**Published:** 2026-07-03

**Authors:** Yaping Song, Haijun Wang, Li Lin, Yibing Cheng, Yuelin Shen

**Affiliations:** 1 Department of Emergency Medicine, Children’s Hospital Affiliated to Zhengzhou University, Henan Children’s Hospital, Zhengzhou Children’s Hospital, Zhengzhou, China; 2 Department of Respiratory Medicine, National Clinical Research Center for Respiratory Diseases, Beijing Children’s Hospital, Capital Medical University, National Center for Children’s Health, Beijing, China

**Keywords:** children, Legionella pneumophila, metagenomic next-generation sequencing, neonate, severe pneumonia, treatment

## Abstract

**Introduction:**

Severe *Legionella pneumophila* (LP) pneumonia is exceedingly rare in children, and clinical data remain scarce.

**Methods:**

We retrospectively analyzed the clinical data of 8 children with severe LP pneumonia diagnosed by metagenomic next-generation sequencing (mNGS) at Henan Children’s Hospital between January 2020 and January 2026.

**Results:**

The cohort comprised 3 males and 5 females with a median age of 74 days (range, 8 days to 9 years); neonates accounted for 50.0% (4/8), and 75.0% (6/8) had no underlying diseases. Six cases were community-acquired and 2 were hospital-acquired. The predominant manifestations were tachypnea/dyspnea (100.0%) and fever (87.5%); neonates presented with lethargy and poor feeding. Complications included respiratory failure (87.5%), multiple organ dysfunction (62.5%), and septic shock (37.5%). Procalcitonin, interleukin-6, and LDH were elevated in all cases, while ALB was uniformly decreased. Bilateral pulmonary involvement was seen in 87.5% on chest imaging. mNGS detected LP in all 8 cases (100%), whereas conventional sputum and blood cultures failed to identify LP in any case; LP was isolated from a surgical pus specimen in only 1 case. Co-infections were identified in 50.0%. All initial empirical regimens failed to cover LP. After mNGS-guided targeted therapy, the fluoroquinolone–rifampin combination (2 cases) achieved complete recovery, while macrolide-based regimens yielded variable outcomes. Overall, 50.0% were cured or improved, while 50.0% died or had treatment withdrawn. LP bacteremia and septic shock were uniformly associated with poor outcomes.

**Discussion:**

Severe LP pneumonia in children predominantly affects neonates and can occur without recognized immunodeficiency. mNGS detected LP in all cases where conventional culture failed. In this small cohort, fluoroquinolone-containing combination regimens were associated with favorable outcomes.

## Introduction

*Legionella pneumophila* (LP) is a Gram-negative intracellular bacterium ubiquitously found in natural and engineered water systems. Humans acquire infection through inhalation of contaminated aerosols, which can result in Legionnaires’ disease, a potentially life-threatening form of pneumonia (Cunha et al., 2016). In adults, LP accounts for 2%–15% of community-acquired pneumonia (CAP) cases, with mortality rates ranging from 4% to 40% (Rello et al., 2024). By contrast, LP pneumonia in children is exceedingly rare and has been reported predominantly as isolated case reports, mostly in immunocompromised hosts (Greenberg et al., 2006). Neonatal infection is particularly uncommon; however, when it does occur, the clinical course is often fulminant, with mortality rates reportedly as high as 50% in infants under one year of age (Perez Ortiz et al., 2021).

The diagnosis of LP pneumonia in children poses substantial challenges. Clinical manifestations are nonspecific and largely indistinguishable from pneumonia caused by other pathogens. LP is a fastidious organism that requires specialized buffered charcoal yeast extract (BCYE) media for culture, with prolonged incubation of 5–7 days, resulting in low detection rates in routine clinical practice. Furthermore, the *Legionella* urinary antigen test detects only serogroup 1 and has limited sensitivity (Pierre et al., 2017). These factors collectively contribute to frequent diagnostic delays and missed diagnoses, during which empirical antibiotic regimens typically fail to provide adequate coverage against LP. In recent years, metagenomic next-generation sequencing (mNGS) has emerged as a valuable tool for infectious disease diagnostics, offering unbiased pathogen detection and the ability to rapidly identify fastidious and rare organisms (Chiu and Miller, 2019; Edward and Handel, 2021). However, data on the application of mNGS in pediatric LP pneumonia remain scarce.

There is a paucity of studies systematically characterizing the clinical features, diagnostic utility of mNGS, and treatment outcomes of severe LP pneumonia in children. We therefore retrospectively analyzed the clinical data of 8 children with severe LP pneumonia diagnosed by mNGS at Henan Children’s Hospital between January 2020 and January 2026, aiming to inform early recognition and optimal management of this rare but serious infection.

## Materials and methods

### Study design and participants

This was a single-center, retrospective case series. We reviewed the clinical records of children hospitalized with severe LP pneumonia at Henan Children’s Hospital between January 2020 and January 2026. The inclusion criteria were: (1) age < 18 years; (2) meeting the diagnostic criteria for severe pneumonia in children (Subspecialty Group of Respiratory, the Society of Pediatrics, Chinese Medical Association et al., 2024); (3) detection of LP in respiratory specimens (sputum or bronchoalveolar lavage fluid (BALF)) and/or blood by mNGS; and (4) availability of complete clinical data. This study was approved by the Medical Ethics Committee of Henan Children’s Hospital (approval NO. 2021-K-L020), and written informed consent was obtained from the guardians of all patients.

### Data collection

The following data were extracted from electronic medical records: (1) demographics and baseline characteristics: sex, age, birth history (gestational age, mode of delivery), underlying diseases, and immune status; (2) epidemiological data: history of exposure to water sources (e.g., showers, humidifiers, swimming pools) and air conditioning systems prior to illness onset; (3) clinical manifestations: presenting symptoms (fever, cough, tachypnea, dyspnea); (4) laboratory findings: complete blood count (white blood cell (WBC) count, neutrophil percentage, lymphocyte percentage), inflammatory markers (C-reactive protein, procalcitonin, interleukin-6), lactate dehydrogenase (LDH), and albumin (ALB), all obtained within 24 hours of admission; (5) chest imaging: chest radiograph and/or computed tomography findings; (6) microbiological data: results of conventional cultures and mNGS, including LP sequence reads, genome coverage, and co-detected pathogens; and (7) treatment and outcomes: antimicrobial regimens and modifications, respiratory support modalities, surgical interventions, length of hospital stay, and final outcomes.

### Microbiological testing

Blood, sputum, and BALF specimens were submitted to the microbiology laboratory of Henan Children’s Hospital for routine culture and identification following standard operating procedures. In parallel, specimens were sent for mNGS testing. DNA was extracted using a standardized protocol, and high-throughput sequencing was performed on the Illumina MiniSeq platform followed by bioinformatic analysis. The mNGS results were jointly interpreted by clinical microbiologists and infectious disease physicians, integrating specimen type, sequence read counts, genome coverage, and clinical context to determine the causative pathogen.

### Statistical analysis

This was a descriptive case series; no hypothesis testing was performed. Continuous variables with uniform reference ranges (CRP, PCT, and IL-6) were expressed as medians with interquartile ranges (M (Q_1_, Q_3_)); for age-dependent parameters (WBC, LDH, ALB, immunoglobulins, and lymphocyte subsets), abnormalities were determined individually using age-specific reference values. Categorical variables were presented as frequencies and percentages (n (%)). Data management and analysis were performed using SPSS version 26.0 (IBM Corp., Armonk, NY, USA).

## Results

### Demographics and baseline characteristics

A total of 8 children with severe LP pneumonia were included, comprising 3 males (37.5%) and 5 females (62.5%). The median age was 74 days (range, 8 days to 9 years); neonates accounted for 4 cases (50.0%, including 1 late preterm infant), infants for 2 (25.0%), and school-age children for 2 (25.0%). Three patients (37.5%) had underlying conditions: prematurity in 1, congenital pulmonary sequestration (intralobar type) in 1, and Down syndrome with congenital heart disease in 1. The remaining 5 (62.5%) had no pre-existing comorbidities or immunosuppression. Six cases (75.0%) were community-acquired and 2 (25.0%) were hospital-acquired. None of the 8 patients had a documented history of exposure to water sources or air conditioning systems (**Table 1**).

**Table 1:** Clinical data of 8 children with severe *Legionella pneumophila* pneumonia.

	Patient 1	Patient 2	Patient 3	Patient 4	Patient 5	Patient 6	Patient 7	Patient 8
Demographics								
Sex	M	F	F	F	M	F	F	M
Age	8 d	8 y 10 m	9 y	28 d	4 m	7 m 25 d	19 d	11 d
Birth history	G5P3, 39^+4^ wk, CS	G1P1, 40 wk, VD	Full-term, CS	G1P1, 36^+4^ wk, CS	G2P2, 39^+2^ wk, VD	G3P3, full-term, CS	G1P1, 41^+4^ wk, VD	G1P1, 40^+3^ wk, CS, grade III meconium-stained amniotic fluid
Underlying conditions	None	Pulmonary sequestration (intralobar)	None	Prematurity	None	Down syndrome; CHD (VSD + ASD)	None	None
Source of infection	CA	CA	CA	CA	HA^a^	CA	CA	HA
Clinical features								
Presenting symptoms	Fever, lethargy, cough, tachypnea	Fever, sore throat, cough, chest pain, hemoptysis	Oliguria, peripheral edema, rhonchi, tachypnea	Fever, poor feeding, facial and limb edema	Dyspnea, fever, cough	Fever, cough, tachypnea	Fever, grunting, tachypnea	Dyspnea, lethargy
Peak temperature (°C)	39.6	39.8	Afebrile	37.7	39.0	39.2	38.6	38.7
Chest imaging	Bilateral patchy opacities	Left lung consolidation, cystic changes, liquefactive necrosis; left pleural effusion	Bilateral patchy opacities; blunting of right costophrenic angle	Bilateral consolidation in all lobes; subcutaneous emphysema on the right	Bilateral consolidation in all lobes; bronchial narrowing	Bilateral pneumonia; obstructive emphysema	Bilateral pneumonia	Bilateral pneumonia; possible right lung abscess
Major complications	RF, bacteremia, shock, MODS	Lung abscess	RF, shock, MODS	RF, bacteremia, shock, MODS	RF, MODS	RF, pulmonary hypertension	RF	RF, MODS
Microbiological findings								
mNGS specimen	Sputum/blood	Sputum	Sputum	Sputum/blood	Sputum	Sputum	Sputum	BALF
LP reads (respiratory/blood)	2,572,366/13976	15,131	3,889	782/8,382	45,522	71,140	15,066	70,685
Co-infecting pathogens (reads)	*A. baumannii* (2254)^b^	None	*S. pneumoniae* (26,406)*, P. aeruginosa* (26,180)	CRAB (470)^b^	None	RSV (280)	None^c^	None
Treatment and outcomes								
Initial empirical therapy	MEM + VAN + IVIG	CRO + VAN	CTX	MEM + LZD	SCF	AMC	CAZ + PCN → MEM + FLC	MEM + LZD
Targeted therapy	Not initiated^d^	AZM+LEV 7d → LEV 14d^e^	Not initiated^d^	EM 4d → AZM 10d → LEV 9d → LEV+DOX 24d → LEV 7d	AZM 5d	LEV+RFP 7d → LEV 4d^f^	RFP+LEV 14d	AZM 14d^g^
Respiratory support	IMV + ECMO	NC	IMV	IMV	CPAP	IMV	CPAP	CPAP
Hospital stay (d)	4	29	3	76	6	52	15	20
Outcome	W/D	Improved	W/D	W/D	W/D	Cured	Cured	Improved

d, days; m, months; y, years; wk, weeks; CS, cesarean section; VD, vaginal delivery; CHD, congenital heart disease; VSD, ventricular septal defect; ASD, atrial septal defect; CA, community-acquired; HA, hospital-acquired; RF, respiratory failure; MODS, multiple organ dysfunction syndrome; mNGS, metagenomic next-generation sequencing; LP, Legionella pneumophila; RSV, respiratory syncytial virus; CRAB, carbapenem-resistant Acinetobacter baumannii; BALF, bronchoalveolar lavage fluid; MEM, meropenem; VAN, vancomycin; IVIG, intravenous immunoglobulin; CRO, ceftriaxone; CTX, cefotaxime; LZD, linezolid; SCF, cefoperazone-sulbactam; AMC, amoxicillin-clavulanate; CAZ, ceftazidime; PCN, penicillin; FLC, fluconazole; AZM, azithromycin; LEV, levofloxacin; RFP, rifampin; DOX, doxycycline; EM, erythromycin; IMV, invasive mechanical ventilation; ECMO, extracorporeal membrane oxygenation; CPAP, continuous positive airway pressure; NC, nasal cannula; W/D, treatment withdrawal/death.

^a^ Patient 5 developed new respiratory deterioration during hospitalization for bacterial pneumonia at another institution and was transferred to our center, where mNGS identified LP as the sole pathogen; classified as HA (referring hospital).

^b^ *A. baumannii* co-infection was confirmed by concurrent positive sputum culture.

^c^ mNGS also detected *E. coli* (816 reads), *A. baumannii* (253 reads), and *Streptococcus mitis* (39,102 reads, oral commensal); none were confirmed by culture and none were classified as co-infecting pathogens.

^d^ The patient was critically ill at the time of diagnosis; targeted therapy was not initiated before treatment withdrawal by the family.

^e^ Left lower lobectomy was performed.

^f^ Concurrent VSD and ASD repair and bronchoscopic lavage were performed; however, the lavage fluid was not sent for microbiological testing.

^g^ Concurrent bronchoscopic lavage was performed.

### Clinical manifestations

All 8 patients presented with tachypnea or dyspnea (100.0%). Fever was present in 7 cases (87.5%), with a median peak temperature of 39.0 °C (range, 37.7–39.8 °C); notably, 1 neonate (Patient 4) had only low-grade fever (37.7 °C), and Patient 3 presented with extrapulmonary symptoms (oliguria and peripheral edema) without fever throughout the clinical course. Cough was observed in 4 cases (50.0%), and chest pain and hemoptysis each in 1 case (12.5%). Neonates predominantly exhibited nonspecific symptoms such as lethargy, poor feeding, and grunting.

Regarding complications, respiratory failure occurred in 7 cases (87.5%), of whom 4 required invasive mechanical ventilation (including 1 receiving extracorporeal membrane oxygenation), 3 received non-invasive ventilation, and 1 was managed with nasal cannula oxygen. Septic shock developed in 3 cases (37.5%), and multiple organ dysfunction in 5 (62.5%), with 3 meeting the diagnostic criteria for multiple organ dysfunction syndrome. LP bacteremia, confirmed by positive blood mNGS, was identified in 2 cases (25.0%). Lung abscess occurred in 1 case (12.5%).

### Laboratory findings

The main laboratory parameters obtained within 24 hours of admission are summarized in **Table 2**. WBC counts ranged from 2.56 to 20.71 × 10^9^/L; based on age-specific reference values, leukopenia was observed in 2 cases (25.0%) and leukocytosis in 1 (12.5%). Neutrophil percentage was elevated in 7 cases (87.5%), and lymphocyte percentage was decreased in 6 cases (75.0%). C-reactive protein was elevated in 7 cases (87.5%), with a median of 73.70 (47.85, 121.40) mg/L; notably, Patient 6 had a normal CRP (2.85 mg/L) despite confirmed LP infection. Procalcitonin was elevated in all 8 cases, though to varying degrees (median 0.56 [0.25, 2.62] ng/mL; range, 0.08–5.26 ng/mL), with only mild elevation in Patients 6 (0.08 ng/mL) and 8 (0.242 ng/mL). Interleukin-6 was elevated in all 8 cases (median 149.10 [41.87, 2476.65] pg/mL; range, 8.36–5000.00 pg/mL). LDH was elevated in 5 cases (62.5%; range, 249.8–702.3 U/L); the 3 neonates (Patients 4, 7, and 8) had values within age-specific neonatal reference ranges. ALB was decreased in all 8 cases (range, 24.2–37.1 g/L).

**Table 2:** Laboratory findings within 24 hours of admission in 8 children with severe *Legionella pneumophil*a pneumonia.

Parameter	Patient 1	Patient 2	Patient 3	Patient 4	Patient 5	Patient 6	Patient 7	Patient 8	Abnormal cases
WBC (×10^9^/L)	2.56↓	4.93	4.77	5.95	20.71↑	4.25↓	10.41	16.37	↓2, ↑1
N (%)	59.8↑	42.0	72.5↑	52.5↑	87.0↑	77.0↑	56.6↑	64.5↑	↑7
L (%)	36.3↓	54.5	21.6↓	39.3	7.2↓	21.0↓	33.6↓	24.0↓	↓6
CRP (mg/L)	105.65↑	52.02↑	46.46↑	77.38↑	161.60↑	2.85	126.65↑	70.02↑	↑7
PCT (ng/mL)	3.02↑	1.44↑	0.28↑	0.738↑	5.260↑	0.08↑	0.387↑	0.242↑	↑8
IL-6 (pg/mL)	5000.00↑	3235.24↑	200.90↑	8.36↑	145.20↑	44.18↑	41.10↑	153.00↑	↑8
LDH (U/L)	702.3↑	257.0↑	373.0↑	249.8	323.2↑	349.6↑	281.0	339.0	↑5
ALB (g/L)	30.7↓	37.1↓	26.8↓	28.9↓	30.2↓	28.2↓	28.9↓	24.2↓	↓8
IgG (g/L)	11.66	11.89	13	9.47	2.68	18.14↑	14.32	11.80	↑1
IgM (g/L)	0.12	0.63	0.55	0.38	0.13↓	0.60	0.48↑	1.10↑	↓1, ↑2
IgA (g/L)	0.01	1.37	1.94	0.07	0.04↓	0.57	0.13↑	0.20↑	↓1, ↑2
CD3+ (%)	63.11	67.30	53↓	82.67	60.27	58.66	85.69	66.42	↓1
CD4+ (%)	46.74	19.78↓	28	61.18	53.58	27.69↓	55.65	44.84	↓2
CD8+ (%)	13.18	42.43↑	21	17.05	5.24↓	30.67↑	27.34	18.77	↓1, ↑2
NK cells (%)	16.78	17.75	23	1.95	4.74	6.60	8.15	12.54	None

↑, above the upper limit of the reference range; ↓, below the lower limit of the reference range. WBC, white blood cell count; N%, neutrophil percentage; L%, lymphocyte percentage; CRP, C-reactive protein; PCT, procalcitonin; IL-6, interleukin-6; LDH, lactate dehydrogenase; ALB, albumin; IgG, immunoglobulin G; IgM, immunoglobulin M; IgA, immunoglobulin A; NK, natural killer cells.

Reference ranges: CRP, < 8 mg/L; PCT, < 0.05 ng/mL; IL-6, < 7 pg/mL. WBC, N%, L%, ALB, LDH, immunoglobulin, and lymphocyte subset reference ranges are age-dependent. Abnormalities (↑/↓) were determined using age-specific reference values for each patient.

### Imaging findings

**Figure 1:**
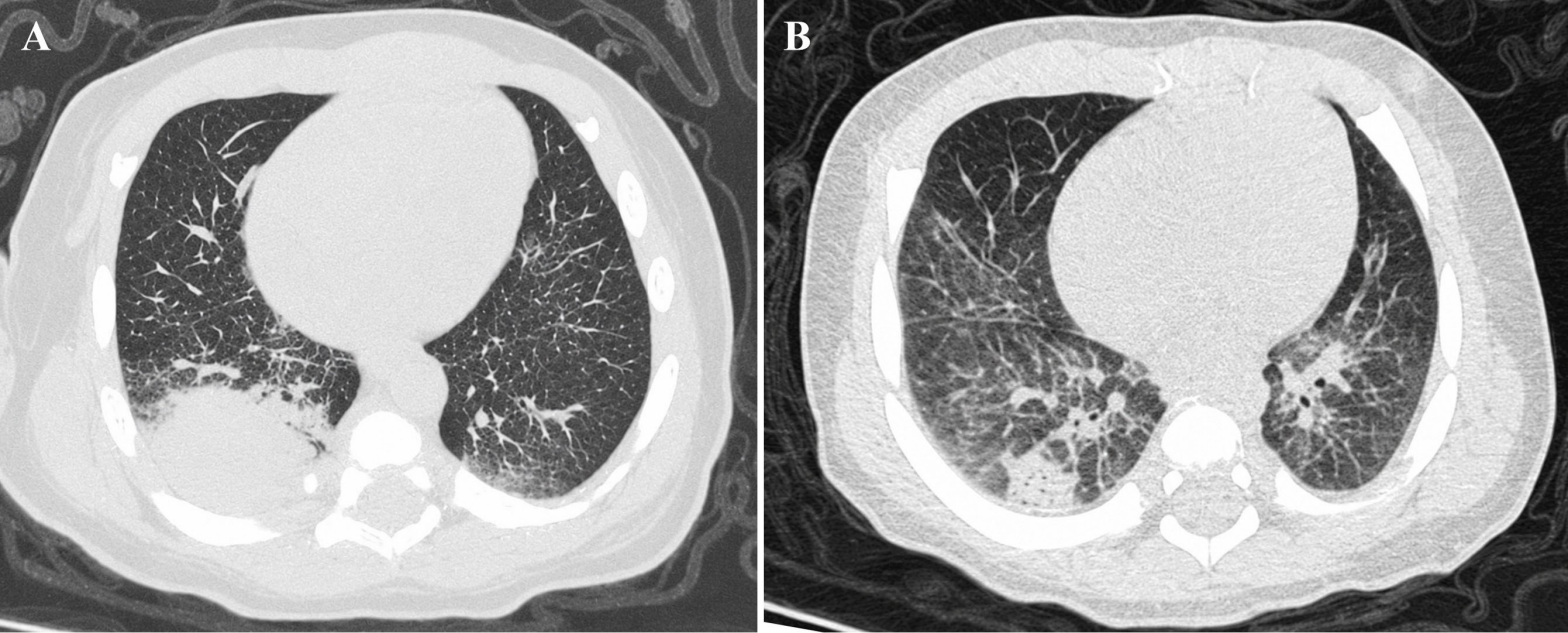
Chest computed tomography of Patient 8 (11-day-old neonate) before and after targeted therapy. **(A)** At admission: bilateral diffuse consolidation with possible right lung abscess formation. **(B)** After 14 days of azithromycin therapy combined with bronchoscopic lavage: marked improvement of the right lower lobe abscess-like lesion.

All 8 patients underwent chest imaging: 6 by computed tomography and 2 (Patients 1 and 3) by bedside chest radiography, as hemodynamic instability during invasive mechanical ventilation precluded transport. Bilateral pulmonary involvement was observed in 7 cases (87.5%) and unilateral (left lung) involvement in 1 (12.5%). The predominant findings were patchy infiltrates; consolidation was present in 3 cases (37.5%), lung abscess formation in 1 (12.5%), and pleural effusion in 2 (25.0%). Rapid radiological progression was noted in several patients (**Table 1**). Representative chest CT images of Patient 8 before and after targeted therapy are shown in **Figure 1**.

### Microbiological findings

mNGS detected LP in all 8 respiratory specimens (sputum in 7 and BALF in 1), with a median sequence read count of 30, 326 (6, 683, 71, 026; range, 782–2, 572, 366). In 2 cases (25.0%), LP was simultaneously detected in blood by mNGS (read counts of 13, 976 and 8, 382, respectively), leading to a diagnosis of LP bacteremia. Conventional sputum and blood cultures failed to identify LP in any case (0/8); LP was isolated from a surgical pus specimen in only 1 patient following lobectomy for lung abscess. Co-infections were identified in 4 cases (50.0%): *Acinetobacter baumannii* in Patient 1, *Streptococcus pneumoniae* and *Pseudomonas aeruginosa* in Patient 3, carbapenem-resistant *Acinetobacter baumannii* in Patient 4, and respiratory syncytial virus in Patient 6.

### Treatment and outcomes

All 8 patients received broad-spectrum β-lactam–based empirical antibiotic therapy upon admission; none of the initial regimens provided coverage against LP or other atypical pathogens. Following mNGS confirmation, targeted anti-LP therapy was initiated in 6 patients. Two patients (Patients 1 and 3) had already developed critical illness at the time of diagnosis, and treatment was withdrawn before targeted therapy could be implemented. The treatment regimens and outcomes were as follows (**Table 1**): Patients 1 and 3 presented with multiple organ dysfunction syndrome and septic shock at diagnosis; both had treatment withdrawn and died. Among the 2 patients receiving macrolide monotherapy, Patient 5 received azithromycin for 5 days with disease progression, ultimately leading to treatment withdrawal and death, whereas Patient 8 received azithromycin for 14 days supplemented with bronchoscopic lavage and was discharged with improvement (Song et al., 2025). Of the 2 patients treated with a macrolide–fluoroquinolone combination, Patient 2 received azithromycin plus levofloxacin for 7 days followed by levofloxacin alone for 14 days, along with left lower lobectomy, and was discharged with improvement; Patient 4 underwent multiple regimen changes including erythromycin, azithromycin, levofloxacin, and doxycycline, but ultimately had treatment withdrawn and died (hospital stay: 76 days). Patients 6 and 7 both received levofloxacin combined with rifampin and achieved complete recovery.

Overall, 2 patients (25.0%) were cured, 2 (25.0%) improved, and 4 (50.0%) died or had treatment withdrawn. The median length of hospital stay was 24.5 days (range, 15–52 days) in the survival group and 5.0 days (range, 3–76 days) in the treatment withdrawal/death group. Both patients with LP bacteremia (Patients 1 and 4) and all 3 patients with septic shock (Patients 1, 3, and 4) had unfavorable outcomes. Both patients who received the fluoroquinolone–rifampin combination were cured (2/2).

## Discussion

LP pneumonia is exceedingly rare in children, with the existing literature consisting predominantly of isolated case reports or small case series, and a systematic characterization of its clinical features remains lacking. The present study retrospectively analyzed 8 children with severe LP pneumonia diagnosed by mNGS, aiming to broaden the current understanding of the clinical spectrum of this disease in the pediatric population.

Neonates accounted for 50.0% (4/8) of our cohort, consistent with the findings of Greenberg et al. (2006), whose systematic review demonstrated that more than half of pediatric *Legionella* infections occurred in infants under one year of age. This age distribution may be attributable to the immunological immaturity of neonates and young infants: LP is an intracellular pathogen whose clearance relies primarily on IFN-γ–mediated activation of macrophages; however, neonates exhibit diminished IFN-γ production and impaired macrophage bactericidal function, rendering them considerably more susceptible to intracellular pathogens than older children (Greenberg et al., 2006; Perez Ortiz et al., 2021). Immunological evaluation revealed abnormalities in several patients, including CD4/CD8 ratio inversion (Patients 2 and 6) and elevated neonatal IgM (Patients 7 and 8). However, as all data were obtained during acute infection, primary immune deficits cannot be distinguished from transient infection-related lymphocyte redistribution without pre-illness baseline data or genetic testing. Notably, 62.5% (5/8) of the patients in our series were previously healthy with no underlying diseases or immunosuppression, whereas the existing literature has reported that pediatric *Legionella* infections predominantly affect immunocompromised children (Greenberg et al., 2006). This discrepancy suggests that immunocompetent children are also at risk of developing severe LP infection, and clinicians should consider this pathogen beyond the immunocompromised population.

Regarding the source of infection, the systematic review by Greenberg et al. (2006) reported that more than half of pediatric *Legionella* cases were hospital-acquired, with nosocomial infections accounting for as many as 77% of neonatal cases; birthing pools have been identified as an important source of nosocomial LP infection in neonates (Collins et al., 2016). To date, no community-acquired outbreaks of *Legionella* in children have been reported, and sporadic community-acquired LP disease in immunocompetent children is considered extremely rare (Greenberg et al., 2006; Perez Ortiz et al., 2021). However, in our cohort, 75.0% (6/8) of cases were community-acquired and only 2 were hospital-acquired, which contrasts with the predominantly nosocomial pattern described in the literature. This discrepancy may be partly explained by the increasing availability of mNGS, which has enabled the identification of community-acquired cases that would previously have been missed by conventional diagnostic methods. No definitive environmental source was identified in any of the 8 patients, likely reflecting the retrospective nature of this study, incomplete documentation of environmental exposures in medical records, and the absence of systematic environmental microbiological investigations. These findings suggest that community-acquired LP infection in children may be more common than previously recognized, although the possibility that some apparently immunocompetent patients harbor subtle immune deficits predisposing to intracellular infection cannot be excluded. The widespread adoption of mNGS may further reshape our understanding of the epidemiology of this disease.

With respect to clinical manifestations, the predominant features were tachypnea/dyspnea and fever, similar to adult LP pneumonia (Cunha et al., 2016; Rello et al., 2024). However, neonates exhibited more atypical presentations including lethargy, poor feeding, and grunting, with one having only low-grade fever and another presenting without fever throughout the course, highlighting the difficulty of early recognition. The high complication rate underscores the severity of this disease, and septic shock was consistently associated with unfavorable outcomes.

Laboratory findings revealed elevated inflammatory markers in most patients, particularly interleukin-6 (reaching as high as 5000.00 pg/mL) and procalcitonin (elevated in all 8 cases), indicating that LP can trigger an intense systemic inflammatory response (Cunha et al., 2016; Rello et al., 2024). However, the degree of elevation varied considerably; Patient 6 had normal CRP and only minimal PCT elevation (0.08 ng/mL), suggesting that a subset of pediatric LP infections may present without marked inflammatory marker elevation. LDH was elevated in most patients (5/8); elevated LDH has been recognized in the adult literature as an important clue for differentiating LP pneumonia from pneumonia caused by other pathogens (Bai et al., 2023), and our findings suggest that this feature may also be applicable in children, although age-specific reference ranges should be considered in neonates. ALB was uniformly decreased (8/8), consistent with adult reports and reflecting protein consumption and capillary leakage in the setting of severe infection (Rello et al., 2024). WBC counts were variable (decreased in 2, normal in 5, elevated in 1), in keeping with the adult literature reporting that WBC counts in LP pneumonia may be normal or only mildly elevated (Cunha et al., 2016; Bai et al., 2023), further suggesting that WBC count has limited diagnostic value in this disease. Taken together, these findings suggest that clinicians should consider LP infection when encountering unexplained severe pneumonia accompanied by elevated LDH, even when conventional inflammatory markers such as CRP are not significantly elevated.

mNGS proved to be of significant diagnostic value in this cohort. Conventional sputum and blood cultures failed to identify LP in any of the 8 cases (0/8); LP was isolated from a surgical pus specimen in only 1 patient. LP is a fastidious organism that requires specialized BCYE media and a prolonged culture period of 5–7 days; most clinical laboratories do not routinely perform *Legionella*-specific cultures, and culture sensitivity is substantially affected by specimen quality and culture conditions (Pierre et al., 2017), resulting in extremely low detection rates. The conventional culture-negative rate in our series was 87.5% (7/8), further highlighting the limitations of traditional methods for diagnosing this disease. In contrast, mNGS detected LP in all 8 cases (100%), independent of culture conditions, and was able to provide results within 24–48 hours, substantially shortening the time to diagnosis (Chiu and Miller, 2019; Edward and Handel, 2021). Moreover, mNGS simultaneously identified co-infecting pathogens (50.0% in this series), including carbapenem-resistant organisms that would not have been identified by targeted respiratory PCR panels, providing valuable information to guide combination antimicrobial therapy. While multiplex PCR panels incorporating *Legionella* are available in some institutions and offer faster turnaround at lower cost, neither *Legionella*-specific PCR nor urinary antigen testing was available at our center during the study period. Furthermore, given the extreme rarity of pediatric LP infection, none of the treating clinicians initially considered *Legionella* in the differential diagnosis, highlighting the particular value of the hypothesis-free nature of mNGS when clinical suspicion is absent.

Regarding treatment, all 8 patients initially received empirical β-lactam–based antibiotic therapy, none of which covered LP—consistent with the pharmacological limitation of β-lactams, which cannot effectively penetrate host cells to target this intracellular pathogen. Currently, there are no large-scale clinical trials or meta-analyses addressing LP pneumonia specifically in children, and treatment strategies are largely extrapolated from adult data. Adult guidelines and consensus statements recommend macrolides or fluoroquinolones as first-line agents (Viasus et al., 2022; Rello et al., 2024). A large retrospective study by Gershengorn et al. (2015), involving 3, 152 adult patients with LP pneumonia across 437 hospitals, found that in-hospital mortality was similar between azithromycin and fluoroquinolone monotherapy, but the combination of azithromycin and a fluoroquinolone was associated with fewer complications. For critically ill or immunocompromised patients, multiple guidelines recommend combination therapy (Viasus et al., 2022; Rello et al., 2024). Regarding treatment duration, mild cases typically require 3–7 days, moderate-to-severe cases 7–14 days, immunocompromised patients up to 21 days, and those with abscess or empyema at least 6–8 weeks (Viasus et al., 2022). In our series, targeted therapy was initiated in 6 patients after mNGS confirmation; 2 patients had already deteriorated critically before diagnosis and treatment was withdrawn. Outcomes varied by regimen: both patients receiving fluoroquinolone–rifampin were cured, whereas macrolide-based regimens yielded variable results. Although the sample size precludes statistical comparison, these findings are consistent with the adult data favoring combination therapy in severe cases (Gershengorn et al., 2015), although no definitive conclusion can be drawn from our limited sample. Nevertheless, combination regimens appear to be a reasonable approach for severe pediatric LP pneumonia.

Regarding drug safety, the use of fluoroquinolones in children has been a subject of concern due to potential musculoskeletal adverse effects. However, a recent meta-analysis found no significant difference in the incidence of musculoskeletal adverse events between fluoroquinolone-treated and non-fluoroquinolone-treated children, with no long-term irreversible damage observed (Wang et al., 2020). The American Academy of Pediatrics has also stated that fluoroquinolone use may be considered in children with severe infections when effective alternatives are unavailable, after careful risk–benefit assessment (Jackson et al., 2016). In our cohort, 3 of 4 patients who received fluoroquinolone-containing regimens had favorable outcomes, and no clinically apparent musculoskeletal adverse effects were observed. With respect to combination therapy, rifampin inhibits bacterial RNA synthesis *via* a mechanism distinct from that of fluoroquinolones and macrolides, and demonstrates potent intracellular bactericidal activity against LP with low minimum inhibitory concentrations (Viasus et al., 2022). *In vitro* studies have shown synergistic killing when rifampin is combined with levofloxacin against intracellular LP (Pedro-Botet and Yu, 2009), providing a rationale for combination therapy in severe cases. In our series, the two patients treated with this combination both achieved complete cure, consistent with the proposed synergistic mechanism. Doxycycline is another anti-*Legionella* agent with excellent intracellular penetration (Cabanilla et al., 2025). In our series, Patient 4 received doxycycline as late salvage therapy, though the disease was already refractory and the outcome was unfavorable. Current evidence supports that short courses of doxycycline do not cause clinically significant dental staining in children under 8 years (Todd et al., 2015); for older children (≥8 years) with severe LP pneumonia unresponsive to first-line agents, doxycycline warrants consideration.

The overall mortality/withdrawal rate of 50.0% is comparable to the approximately 50% reported by Perez Ortiz et al. (2021) for neonatal LP pneumonia. LP bacteremia and septic shock were consistently associated with poor outcomes, highlighting the need for early diagnosis and prompt targeted intervention.

This study has several limitations. First, as a single-center retrospective study with only 8 cases, the generalizability of the conclusions is limited. Second, precise data on the interval from disease onset to mNGS diagnosis were not available, precluding quantification of the contribution of mNGS to reducing diagnostic delay. Third, comparisons of treatment efficacy across regimens were limited by the small sample size and could not be subjected to statistical analysis. Fourth, whole exome sequencing was not performed to exclude underlying inborn errors of immunity, which limits our ability to definitively characterize host susceptibility in this cohort. Additionally, some patients had treatment withdrawn by their families rather than experiencing treatment failure, potentially underestimating the actual therapeutic efficacy. Future multicenter studies with larger sample sizes are needed to further define the optimal diagnostic and treatment strategies for pediatric LP pneumonia.

In conclusion, this case series demonstrates that severe LP pneumonia in children predominantly affects neonates and young infants and can occur in the absence of recognized immunodeficiency. Clinical presentations are nonspecific, particularly in neonates, underscoring the need for heightened clinical awareness. mNGS enabled pathogen identification in all 8 cases where conventional culture failed, suggesting its value in diagnosing this rare infection. In our limited cohort, fluoroquinolone-containing combination regimens were associated with favorable outcomes; however, given the small sample size, further studies are needed to define optimal treatment strategies for pediatric LP pneumonia.

## Data Availability

The raw data supporting the conclusions of this article will be made available by the authors, without undue reservation.
